# Configurable 3D Printed Microfluidic Multiport Valves with Axial Compression

**DOI:** 10.3390/mi12101247

**Published:** 2021-10-14

**Authors:** Juliane Diehm, Verena Hackert, Matthias Franzreb

**Affiliations:** Institute of Functional Interfaces, Karlsruhe Institute of Technology, Hermann-von-Helmholtz-Platz 1, 76344 Eggenstein-Leopoldshafen, Germany; juliane.diehm@kit.edu (J.D.); verena.hackert@kit.edu (V.H.)

**Keywords:** rotatory valve, rapid prototyping, polyjetting, digital light processing (DLP), sealing

## Abstract

In the last decade, the fabrication of microfluidic chips was revolutionized by 3D printing. It is not only used for rapid prototyping of molds, but also for manufacturing of complex chips and even integrated active parts like pumps and valves, which are essential for many microfluidic applications. The manufacturing of multiport injection valves is of special interest for analytical microfluidic systems, as they can reduce the injection to detection dead volume and thus enhance the resolution and decrease the detection limit. Designs reported so far use radial compression of rotor and stator. However, commercially available nonprinted valves usually feature axial compression, as this allows for adjustable compression and the possibility to integrate additional sealing elements. In this paper, we transfer the axial approach to 3D-printed valves and compare two different printing techniques, as well as six different sealing configurations. The tightness of the system is evaluated with optical examination, weighing, and flow measurements. The developed system shows similar performance to commercial or other 3D-printed valves with no measurable leakage for the static case and leakages below 0.5% in the dynamic case, can be turned automatically with a stepper motor, is easy to scale up, and is transferable to other printing methods and materials without design changes.

## 1. Introduction

3D printing became a game-changer for the manufacturing of microfluidic chips [1,2]. It not only simplifies manufacturing processes, but also enables the usage of microfluidic systems for a wide range of applications by combining increased configurability with high economic efficiency, especially for the manufacturing of prototypes [1,3,4]. While 3D printing was initially mainly used for the fabrication of molds and simple parts, complex chip designs and active components such as valves and pumps, which are essential for many microfluidic applications [5], can also be produced using 3D printing nowadays [3,6].

For the 3D printing of microfluidic valves, two core approaches exist: for applications that allow an implementation with 2/2 way valves or a combination thereof, there is a broad spectrum of developed mechanisms, including but not limited to 4D printing, and new methods tailored to specific applications are constantly being further developed. 4D-printed valves react to an external stimulus like magnetic fields [7,8], pH [9], or temperature [10] and thus change their switch position. Other mechanisms include membrane-like valves [11,12,13] and slope valves [14]. Further approaches can be found in numerous review articles [15,16,17].

**Figure 1:**
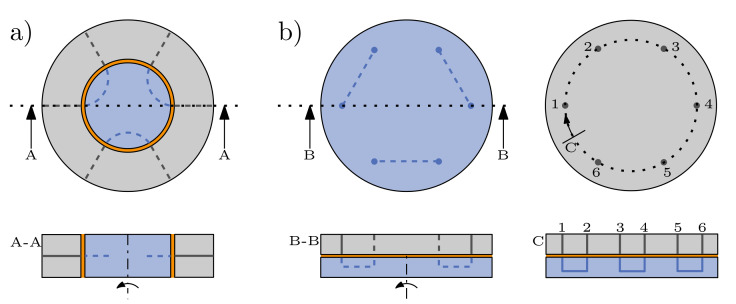
Schematic top and section view of a 6-port injection valve with radial compression (**a**) and axial compression (**b**). Rotor is shown in blue, stator in gray, and sealing gap in orange. For the axial compressed valve, an additional circular sectional view (C) is given, displaying interconnections of rotor and stator along dotted circle. To achieve tightness, additional parts are needed for compression with axial approach.

In applications where 2/2 way valves cannot be used, e.g., when connections between different channels are to be changed, the rotor-stator principle, which is also utilized in many commercial valves, is employed: Morioka et al. developed a 3D-printed stator in combination with a polydimethylsiloxane (PDMS) microfluidic chip and improved the response time and minimized band-broadening effects in a flow injection analysis system [18]. Su et al. developed a 3D-printed flow injection system for coupling with ICP-MS [19], Chan et al. designed pump and valve systems for point-of-care diagnostics [20], and Munshi et al. designed a valve for sample injection volumes as low as 195 nL [21]. All those systems use radial compression of rotor and stator (only Su et al. used a combination of radial and axial compression by a slanted sealing surface [19]) in contrast to commercial valves, like Rheodyne valves, that are usually compressed in the axial direction [22,23,24]. The difference between both approaches is illustrated in Figure 1. For the radial approach, the cylinder jacket surface of the rotor is the sealing surface and the compression of rotor and stator, and thus, the sealing is achieved by choosing the right fit for the manufacturing process. For the axial approach, the sealing surface corresponds to the base plate of rotor and stator. Although this design requires additional parts to compress rotor and stator, there are many advantages that accompany external compression: the compression can be adjusted, e.g., adapted to increasing wear-effects, it is easier to combine different materials as a precise fit is not as important as in the radial variant, and external sealing elements can easily be used to improve the sealing performance of the system.

In some cases, especially for applications requiring numerous switching operations between different valve positions, commercial axial compressed valves were integrated into 3D-printed chips, e.g., for lab-on-valve systems: Mattio et al. developed a lab-on-valve platform with a 3D-printed chip and demonstrated its applicability for the detection of cadmium and lead in water [25]. Cocovi–Solberg et al. used a similar system for various biochemical assays [4]. In all cases, a 3D-printed stator was combined with a commercial rotor system, utilizing the rotor seal for sealing the system. While this approach is straightforward, it still has drawbacks since the rotor has to be used as is, thus limiting designs to available configurations. In our opinion, this does not exploit the great potential of 3D printing entirely; at the same time, it is not trivial to design suitable 3D-printed valves for applications where the valves need to be turned frequently and have high requirements on tightness and wear-resistance.

In this work, we adapted and tested the axial compression approach for 3D printing using polyjetting and digital light processing (DLP) as printing techniques. In total, six different sealing concepts are tested. The tightness of the resulting system is evaluated using three different techniques. This approach enables the integration of 3D printable components like merges, diverges, and mixers in the stator as well as the rotor, thus we hope to further increase the impact of 3D printing on microfluidic chip fabrication and expand the field of applications.

## 2. Materials and Methods

### 2.1. Valve Design and Fabrication

**Figure 2:**
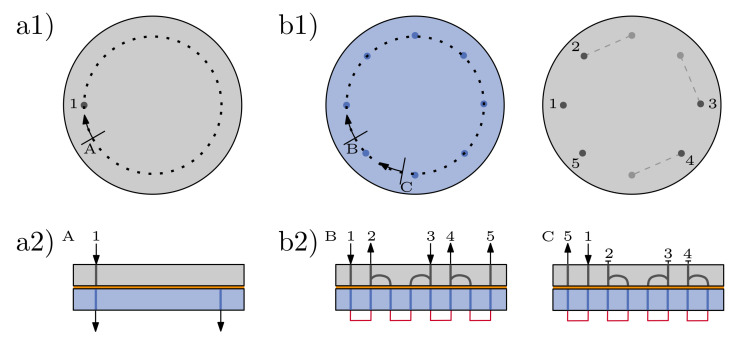
Schematic views of test setups. Stator is depicted in gray, rotor in blue. (**a1**) Top view of stator and (**a2**) circular sectional view of a test valve with one inlet and two outlet options. (**b1**) Top view of rotor and stator of an upscaled valve; rotor features 8 inlet/outlet connections, stator features 5 with additional merging/diverging channels integrated into stator plate. (**b2**) Circular sectional views of two connection schemes of upscaled valve. In both cases the inlet/outlet connections of rotor are short-circuited (no external connections). Left-hand side shows a case where all in- and outlets are used, and on the right-hand side, only one in- and outlet are connected.

The valves were designed with the computer-aided design (CAD) software Inventor Pro 2020 (Autodesk Inc., San Rafael, CA, USA) and exported as stl-files for 3D printing. A 3/2 way valve (referred to as test valve) was designed to evaluate the different sealing concepts. The basic flow paths in rotor and stator can be seen in Figure 2a. All flow channels had a diameter of 800 μm. After a first assessment of different sealing concepts, an upscaled 13/4 way valve was designed based on the same concept; a basic connection scheme is given in Figure 2b. In this study, two different 3D printing techniques, polyjetting and DLP printing, were used to evaluate six different sealing concepts, which are explained in more detail in the following subsections.

#### 2.1.1. Polyjet 3D Printing

For polyjetting an Object EDEN 360V (Stratasys, Rechovot, Israel) was used. VeroClear was selected as printing material and SUP705 as support material, and both are produced by Stratasys as well. After printing, the parts were mechanically cleaned from support structures, and then put in a 2 M sodium hydroxide (Merck KGaA, Darmstadt, Germany) solution for 1 h to dissolve remaining support material inside the microfluidic channels. Subsequently, the parts were cleaned with deionized water and dried before further usage.

#### 2.1.2. DLP 3D Printing

For DLP printing, an ASIGA Max X35 (385 nm) (Asiga, Alexandria, Australia) was used. Basic valve parts were printed with BV-007 microfluidic resin (Young Optics, Jena, Germany). For 3D-printed sealing elements, a highly fluorinated methacrylate based resin was prepared according to Kotz et al. [26] with Fluorolink MD700 (Solvay speciality polymers italy, S.p.A., Bollate, Italy), 5 mg/mL phenyl-bis-(2,4,6-trimethylbenzoyl)-phosphinoxid (Sigma-Aldrich, St. Louis, MO, USA) and 0.2 mg/mL Sudan Orange G (Sigma-Aldrich, St. Louis, MO, USA). After printing, the parts were cleaned with isopropanol in an ultrasonic bath for 5 min twice. Printed support structures were removed and the parts were postcured with an Otoflash G171 polymerization device (NK Optik GmbH, Baierbrunn, Germany). Independent of the used printer, 1/4-28” UNF threads were drilled after the printing process for external fluid connections with flangeless fittings, as this proved to be more durable than direct printing in previous work.

#### 2.1.3. Sealing Concepts

**Figure 3:**
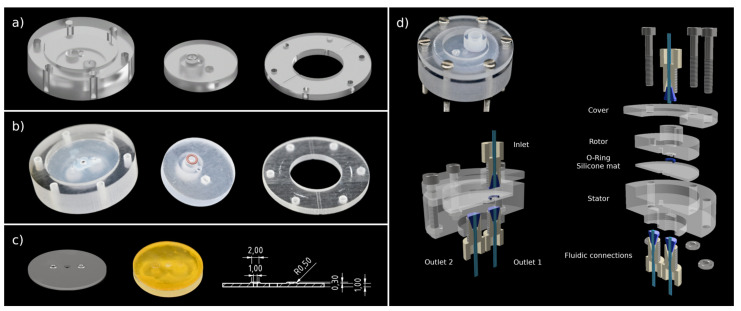
Design and implementation of test valve: (**a**) CAD drawing of stator, rotor, and cover; (**b**) 3D-printed rotor, stator, and cover: sealing area of the rotor is covered with a silicone mat, and an o-ring is placed in respective groove in rotor. Parts were printed with polyjetting; (**c**) from left to right: CAD drawing of 3D-printed sealing mat with integrated o-ring-like structures, 3D-printed sealing mat, technical drawing of the sealing mat; (**d**) top-left: assembled 3D-printed test valve; bottom-left and -right: section and exploded view of a CAD drawing of sealing concept 5 showing placement of respective sealing elements.

All tested valves consist of three basic parts: a stator, a rotor and a cover used to compress the rotor and stator in axial direction with screws. A CAD-drawing of those parts as well as an example of the printed parts are given in Figure 3a,b. Depending on the respective concept, the parts were used as is, postprocessed, or additional sealing elements were inserted. For postprocessing, the 3D-printed parts were polished with sanding sponge (3M Softback Sanding Sponge Grade Micro Fine, 3M, Saint Paul, MN, USA) attached to an insert of a rotatory tool (Dremel 4000, Dremel Europe, Breda, The Netherlands). O-rings (FKM, 3 × 1 mm, Dichtomatik, Hamburg, Germany), a silicone mat (1 mm, 40${}^{\circ }$ shore A, exact plastics GmbH, Bröckel, Germany), DLP printed sealings mats and combinations hereof were evaluated regarding their functionality as sealing elements. The 3D-printed sealing mats had integrated o-ring-like structures, as can be seen in Figure 3c. Loctide 401 (Henkel AG & Co. KGaA, Düsseldorf, Germany) was used to glue the respective sealing mats to the printed parts. In total, six different sealing concepts were tested; an overview is given in Table 1. Figure 3d shows an assembled test valve as well as a section and exploded view displaying the configuration of sealing concept 5 in detail. Stl-files of the different test valves can be found in the Supplementary Materials. To evaluate the performance of the different concepts, sealing tests (see Section 2.2) as well as light microscope imaging of sealing surfaces and o-rings were performed. Microscope images were obtained using a Leica DM 2500M microscope (Leica Microsystems GmbH, Wetzlar, Germany) in dark field mode.

**Figure 4:**
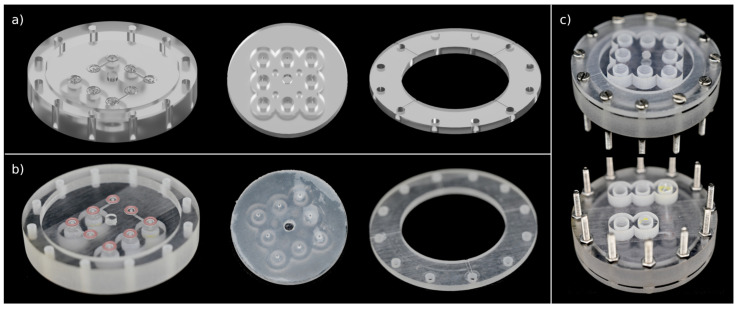
Design and implementation of the upscaled valve: (**a**) CAD drawing of stator, rotor, and cover; (**b**) 3D-printed rotor, stator, and cover: sealing area of rotor is covered with a silicone mat, and o-rings are placed in respective grooves in stator. Parts were printed with polyjetting; (**c**) top and bottom view of assembled upscaled valve.

Concept 5 was chosen for the design of the upscaled valve using polyjetting for printing. The upscaled valve has 8 instead of 1 intravalve connections, and the sealing area is 2.7-times larger compared to that of the test valve. The single components and the assembled valve can be seen in Figure 4.

### 2.2. Sealing Tests

To evaluate the tightness of the 3D-printed system, sealing tests with three different setups were performed. The details are given in the following subsections.

#### 2.2.1. Static and Dynamic Sealing Test

For an initial static sealing test, the test valve was assembled (see Figure 3d) and the screws were tightened such that the resulting torque needed to turn the valve was between 80 and 100 Ncm. The inlet of the test valve was then connected to an Äkta Purifier system (Cytiva Europe GmbH, Freiburg, Germany). The tightness of the valve was tested at different pressures, and the pressure was adjusted by increasing the flow of water through the valve with the pump of the Äkta. Each pressure was kept for 10 min before increasing to the next level (approximately 1 bar steps). The leakage of the valve was determined by optical examination, which was possible as all valve parts were printed with transparent materials. This method is less accurate than the other methods used, but nonetheless gives a first impression whether a concept is suitable at all. The experiment was stopped if a maximum pressure of 15 bar was reached without the observation of leakage. This test was repeated with test valves of all sealing concept configurations.

In the next step, the abrasion of the respective sealing elements was tested with the dynamic sealing test. This experiment was performed with all test valves that were tight at a pressure of 15 bar in the previous static test. The setup was as described before, but the pressure was kept constant at 8 bar, and every 30 s the valve was manually switched from outlet 1 to outlet 2 (compare Figure 3d) until 5 complete rotations were reached. The flow was stopped during the rotation.

#### 2.2.2. Influence of Compression and Pressure

For further tests, to determine the influence of compression and pressure on the sealing performance, the upscaled valve was used. The valve was connected to an OB1 MK3+ (4 channels, −1000–6000 mbar) microfluidic flow controller (Elvesys, Paris, France) as depicted in Figure 2(b2) (left-hand side). The leakage of the system was determined by weighing the valve before and after the experiment with an analytical balance (Adventurere AX224, OHAUS Corporation, Parsippany, NJ, USA). To compensate for swelling effects of the 3D-printed material, the system was initially flushed with water for 1 h at a pressure of 500 mbar. Firstly, the pressure provided by the microfluidic flow controller was kept constant at 500 mbar, while the axial compression of the valve was varied so that the required torque needed to turn the rotor was between 20–100 Ncm. The valve was rotated every 30 s by 90${}^{\circ }$ for 10 min using a stepper motor (PD60-3-116-TMCL, TRINAMIC Motion Control GmbH & Co. KG, Hamburg, Germany). The script used to turn the motor with the respective settings can be found in the Supplementary Materials. The experiment was run for 10 min and was performed in triplicates. As a control, the same experiment was performed without switching the valve.

In a second test series, the torque was kept constant at 30 Ncm and the pressure was varied between 50 and 1000 mbar. The valve was again turned by 90${}^{\circ }$ every 30 s, and the experiment was run for 10 min and performed in triplicates.

#### 2.2.3. Interplay between Valve and Flow Source

In a third sealing test, the valve’s performance was evaluated using flow sensors (SLI-1000, Sensirion AG, Stäfa, CH) connected to both the inlet and the outlet of the system. The setup was as depicted in Figure 2(b2) (right-hand side) using only one inlet and outlet port of the valve stator. The microfluidic flow controller operated at 350 mbar as well as the piston pump P9 A of an Äkta Pure (Cytiva Europe GmbH, Freiburg, Germany) at a flow rate of 200 μL/min were used as pressure sources. The flow rates were recorded for 10 min, while the valve was again turned by the stepper motor every 30 s. For comparison, the flow rates at the inlet and outlet of the column valve of the Äkta were measured. The column valve was switched every 30 s between position 1 and 2. Afterwards the flow rate measurements were divided into 30 s intervals for data evaluation, with one switching process taking place in each interval. To compensate for any differences of the flow rate measurements between the used flow rate sensors, both flow rate sensors were connected in series and the flow through both sensors was measured for 10 min. The experimental results were adjusted according to the observed difference as described by Equation (1), with $V_{inlet,E}$ and $V_{outlet,E}$ being the total measured volume that passed through the inlet and outlet flow sensor, respectively, in a 30 s interval during an experiment and $V_{inlet,S}$ and $V_{outlet,S}$ being the total volumes measured with the respective sensors during the sensor comparison.

(1) $$Leakage\left(\%\right)=\left(\frac{V_{inlet,E}-V_{outlet,E}}{V_{inlet,E}}-\frac{V_{inlet,S}-V_{outlet,S}}{V_{inlet,S}}\right)\cdot 100\%$$

## 3. Results and Discussion

### 3.1. Evaluation of Sealing Concepts

**Figure 5:**
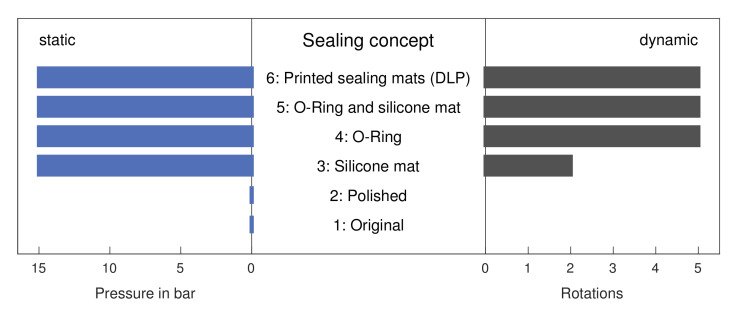
Comparison of tested sealing concepts; (**left**) pressure stability of respective sealing concepts (static sealing test); (**right**) influence of number of rotations on tightness of test valves (dynamic sealing test).

To identify suitable sealing concepts the valve was first treated as a static system as those are less complex to seal than dynamic ones [27]. The tightness of the different systems in dependence of the applied pressure is given in Figure 5 on the left side. The first two sealing concepts, which do not use specific sealing elements, leaked immediately, even when no measurable pressure was applied. This observation was made regardless of the used printing technique and is caused by the rough surface of the 3D-printed parts which always leads to a small gap between rotor and stator, even if both parts are strongly compressed [21]. This result is consistent with reports from literature, where either lubricants like vaseline [20], teflon spray [19] or teflon wrap [21] were used or only the rotor was 3D-printed with an elastic PDMS stator that ensured sealing [18,28]. Further details such as microscope images regarding the structure of the sealing surfaces are given in Figure S1 in the Supplementary Materials. All valve concepts using additional sealing elements were tight up to a pressure of at least 15 bar, regardless of the design, which is to the best of our knowledge the highest pressure applied to any 3D-printed microfluidic valve so far and clearly shows the benefit of the external sealing elements used in this study.

Based on these results, the dynamic test was performed with designs 3–6 only to compare the wear resistance of the different sealing elements. Figure 5 (right-side) shows designs 4, 5, and 6 showed unchanged tightness after 5 rotations, and it was only with design 3, which uses a silicone mat, that leakage occurred after 2 rotations. Hence, the silicone mat can compensate for the roughness of the 3D-printed parts in the static sealing test, but cannot be used for real-world operation. Thus, o-ring-based concepts seem to be the most promising. In our tests, we neither observed any difference between a classic o-ring and the 3D-printed o-ring-like structures, nor between the two printing techniques, even though the printers have different resolutions and tolerances and different printing materials were used. Consequently, the presented concept enables not only a simple transfer to new printing methods or materials without the need for preliminary test to determine fits and tolerances, but it also allows for an easy usage of a variety of sealing elements, in contrast to the traditional radial concept, where rotor and stator are typically transitionless or press-fitted, which makes it complicated to insert additional elements into the sealing gap.

Long-term tests showed that the reusability of o-rings of sealing concept 5 is superior to sealing concept 4, as the wear of the o-rings on the rough surface of the 3D-printed parts is higher compared to that of the smoother silicone mat. Microscope images of o-rings after 20 rotations with and without a silicone mat on the opposite sealing surface show only slight deformations of the o-rings with the silicone mat and huge abrasions without. The respective images can be found in Figure S2 of the Supplementary Materials. Therefore, concepts 5 and 6 show the best performance for long-term applications, and the 5th concept was chosen for further studies. In this particular case, concept 6 was not considered because the building platform of the DLP printer is too small for the upscaled valve. In principle, however, this approach is just as promising and has similar performance. The upscaled system is presented in Figure 4. In the static and dynamic sealing test, this valve shows identical performance to the test valve. For this system, the dynamic sealing test was repeated with 50 rotations without the occurrence of leakage, indicating that the system is suitable for long-term applications.

### 3.2. Axial Compression and Pressure

**Figure 6:**
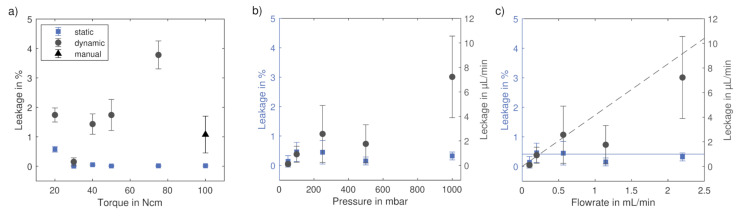
Influence of axial compression and pressure on leakage; (**a**) leakage stream of upscaled valve in % in dependence of torque required to rotate valve. Experiments were performed at a pressure of 500 mbar in triplicates; (**b**) influence of applied pressure on leakage stream of upscaled valve. Experiments were run at a torque of 30 Ncm and performed in triplicates; (**c**) results from (**b**) shown in dependence of flow rate instead of pressure. Blue line shows maximum relative dynamic leakage, and dashed gray line maximum absolute dynamic leakage under experimental conditions.

In the previous test, the flow was turned off during the switching process. This is not always practicable, and thus another test was performed with continuous flow. Simultaneously, the influence of the axial compression on the valve’s tightness was evaluated. The results are given in Figure 6a. The leakage was calculated by dividing the measured mass gain of the valve by the overall mass flow through the valve. It was below 0.06% of the system flow in the static case with a torque ranging from 30–100 Ncm. These values are in the range of measurement inaccuracy (the average standard deviation of weighing the valve in relation to the overall mass flow is 0.04% for all performed experiments), confirming the tightness of the system in the static case and the results from the previous tests. The leakage stream increased above 0.5% at a torque of 20 Ncm, indicating that the contact pressure is too low to ensure tightness.

In the dynamic case, the leakage has a minimum at 30 Ncm with an average leakage of 0.15%. This corresponds to a leakage of 0.15 μL/min which is in the same order of magnitude as the leakage of commercial valves (e.g., leakage < 0.1 μL/min for Äkta Valve INV-907 [29]), however it has to be kept in mind that commercial valves usually are tested at higher pressures. The leakage increases for lower torques due to insufficient compression, while it increases at higher torques due to the decreasing accuracy in positioning by the motor (the probability of the motor to miss steps and thus turn less than 90${}^{\circ }$ increases with increasing torque). Theoretically, one would expect an increasing sealing effect with increasing contact pressure, but here the opposite is the case. To verify that this effect is indeed caused by the inaccuracy of the motor, an experiment with a manual operation at a torque of 100 Ncm was performed. Due to the increased precision in manual positioning at high torques, the leakage is lower compared to that of positioning with the motor. However, the switching time is about 8 times higher (approximately 1 s during manual operation and 0.125 s using the motor according to manufacturer specifications). This explains why the leakage is still higher with manual operation at 100 Ncm compared to that of operation with the motor at 30 Ncm.

These dependencies in combination with the complete tightness of the system in the static case lead to the assumption that leakage only occurs during the switching process. This conclusion is further confirmed by the results shown in Figure 6b,c, where in both cases the relative and absolute leakage is shown in dependence of pressure and flow rate, respectively. The relative leakage is below 0.5% for all cases, whereas the absolute leakage seems to increase with increasing pressure/flow rate. As the switching time is constant, it is reasonable that the relative leakage is approximately constant while the absolute leakage increases. The expected leakage streams during switching if the valve had no sealing effect during rotation can be calculated by multiplying the overall switching time with the respective flow rate. In Figure 6c, those expected values are given in a solid blue line for the relative leakage and in a dashed gray line for the absolute case. The mean of the measured values is about 75% of the predicted value for total leakage during switching processes; taking into account the standard deviation, the values are in the same range.

Dynamic sealing problems cannot be sealed 100% [27,30]. A relative leakage of less than 0.5% is small compared to uncertainties in common detection methods like UV/Vis spectroscopy, where the uncertainty is in the single-digit percentage range [31], and it is thus questionable if it would be noticeable in an experiment. At the same time, the case tested here represents an extreme test scenario in which the valve was switched more frequently than in most common applications; when used as an injection valve, for example, only one switching process is required per experiment. A visible leakage was only observed at a torque of 80 Ncm and a pressure of 1000 mbar, respectively, even then, only a thin film of liquid was visible in the sealing gap, which wouldn’t be noticeable if the parts weren’t transparent.

### 3.3. Interplay between Valve and Flow Source

In former studies of 3D-printed radial valves, water leakage tests were performed but it is not specified how the systems tightness was determined [18,28], making the results hard to compare. In addition the tightness of one particular system may also be subject to the respective experimental setup, thus it is not common in literature as well as in manufacturer information to give the intrinsic leakage streams for dynamic sealing systems like valves. Nonetheless, it is of interest how different systems react during a valve switching process. For this purpose, the flow rate at the valve in- and outlet were measured during switching. This method is less accurate for the determination of leakage compared to weighing, but as the measurement can be done in-line interesting insights in processes during the switching can be obtained. In this study three different setups were compared which used either a microfluidic flow controller or a piston pump as flow source, combined with the 3D-printed valve or the column valve of an Äkta.

**Figure 7:**
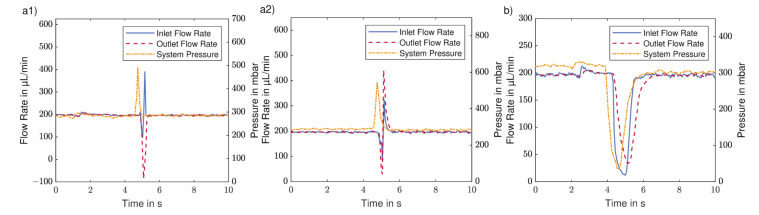
Flow rate profiles at valve in- and outlet and system pressure; (**a1**) 3D-printed valve with piston pump, not tight case; (**a2**) 3D-printed valve with piston pump, tight case; (**b**) Äkta column valve with piston pump. Due to different accuracies of time stamps of used flow and pressure sensors, there is a maximum possible offset between shown pressure and flow rate curves of 0.5 s.

The results are given in Figure 7, showing exemplarily the inlet flow rate in a solid blue line, the outlet flow rate in the red dashed line and the system pressure in a dashed dotted yellow line for the different setups. Due to different accuracies of the time stamps of used flow and pressure sensors, there is a maximum possible offset between the shown pressure and flow rate curves of 0.5 s. For the 3D-printed valve, independent of the used pressure source, two different cases can be observed for the switching process, which are depicted exemplarily in Figure 7(a1,a2) for the valve connected to the piston pump. The corresponding graphs for the microfluidic flow controller can be found in the Supplementary Materials (Figure S3). In a1, both flow rates drop at the beginning of the switching process, and afterwards, the inlet flow rate rises to almost 350 μL/min after approximately 200 ms. After 600 ms, both flow rates recover their initial level. The pressure rises above 400 mbar before dropping to the initial value again. In a2, both flow rates perform a double peak during the switching process, first dropping below 50 μL/min, then rising above 300 μL/min. The pressure rises above 500 mbar before dropping to the initial value again. Figure 7b shows that both flow rates as well as the pressure drop to a value below 50 μL/min or 50 mbar, respectively, before rising to the original value for the column valve connected to the piston pump.

When comparing Figure 7(a1) to (a2), both curves start similarly, but then in a1 the inlet flow rate increases without the outlet following, indicating leakage. This is probably caused by the built-up pressure caused by the piston pump. For a tight system, a behavior as depicted in Figure 7(a2) is expected. During switching, all flow paths are blocked, and thus the system pressure increases. After the flow channels are connected again, the flow can build up, the flow rates increase due to the increased pressure, thus decreasing the pressure until both flow rates and pressure drop to the original value. In contrast, for the Äkta system the pressure drops during the switching process. As the pressure sensor is located directly after the piston pump, the only plausible explanation for this is that the pump stops pumping during valve switching. This also causes both flow rates to drop during the switching processes without the occurrence of an overshoot of flow or pressure. While this is certainly favorable to increase the system’s tightness during the switching process as well as to prevent pressure or flow spikes after the switching, it also leads to a longer switching time. In this experiment, the switching time is two-times higher compared to that of the 3D-printed system.

Both scenarios depicted in Figure 7(a1,a2) were observed for the 3D-printed valve in dependence of the current switching position but independent of the used flow source. In conclusion, leakage occurs sporadically for the 3D-printed valve, indicating that the used 3D printers have a noticeable manufacturing tolerance. Nonetheless, previous experiments showed that the absolute leakage is at a very low level that won’t interfere with the majority of applications. Even if it would be relevant in some cases, it can be prevented by turning the pump off during switching, as it is done by the Äkta system.

**Figure 8:**
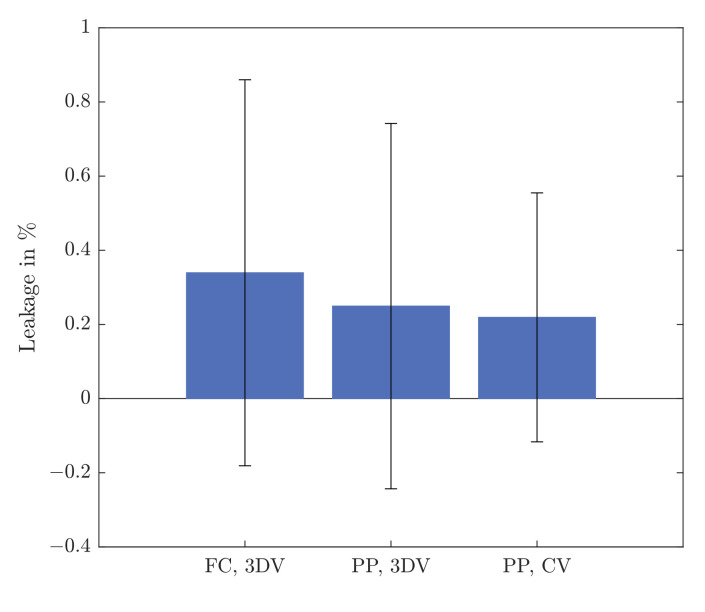
Difference in integrated in- and outlet flow for three tested setups. FC: microfluidic flow controller; 3DV: 3D-printed valve; PP: piston pump; CV: column valve of Äkta system.

Figure 8 shows a comparison of observed leakages over all switching processes for the different setups. All mean values are in the range of 0.2–0.35%. The standard deviation is ±0.5% for both experiments with the 3D-printed valve, and a little less (±0.3%) for the experiment with the column valve. This is in accordance to the manufacture’s specifications, where the repeatability of the sensors is stated to be below 0.5% of the measured value. The mean as well as the standard deviation of the 3D-printed valve is slightly larger compared to the value of the Äkta system in both tested cases, which is plausible due to higher manufacturing tolerance of 3D printing different switching scenarios that were observed with leakages occurring sometimes but not always, as discussed previously. This was not observed for the Äkta system, as the flow was stopped during the switching in this case. The difference of the leakages are too small to distinguish any differences between the setups, as all mean values as well as zero (no leakage) are in the range of the observed standard deviations. This shows that within the accuracy of our measurements the sealing of the 3D-printed valve is comparable to a commercial one. In addition, this experiment gives a good insight in the processes taking place during switching and can be used as a starting point for further optimization of the 3D-printed valve system.

Table 2 summarizes the advantages and disadvantages of different valve manufacturing approaches and lists the features of applications that the manufacturing approaches are suitable for.

## 4. Conclusions

In this study, we developed and tested an axially compressed, 3D-printed microfluidic multiport valve. The valve’s tightness was evaluated with three methods: optical examination was used for a first qualitative analysis of the systems. Weighing proved to be the most accurate method for quantitative studies. Flow rate measurements are less sensitive for quantitative measurements if only slight differences in flow rates can be observed, but can provide additional insights into the processes during switching. The designed valve had a comparable performance to that of radial 3D-printed multiport valves and the commercial valve system evaluated in this study, with no measurable leakage for the static case and leakages below 0.5% in the dynamic case. Six sealing configurations with additional sealing elements were examined. A concept using o-rings and a silicon sealing mat as external sealing elements showed the best performance, especially for long-term applications. The proposed approach could easily be transferred to other 3D printers using different printing materials without the need for preliminary experiments or the adjustment of the fit of rotor and stator. The systems performance was optimized through the adaption of the axial compression in a setup where the valve was switched automatically with a stepper motor. In addition, the valve can easily be scaled up in regard to sealing surface and number of channels and is highly costumizable, as the rotor as well as the stator are 3D printed. Thus, additional elements like merges, diverges, microfluidic mixers, or external fluidic connections can directly be integrated into all fluidic paths of the valve, minimizing the dead volume of the corresponding setup. This contributes to lab-on-valve applications where the valve is designed according to the respective application and not vise versa, increasing the versatility of 3D-printed microfluidic applications.

## Figures and Tables

**Table 1:** List of tested sealing concepts.

Sealing Concept No	3D Printer	Sealing Elements
1	Polyjet/DLP	-
2	Polyjet	-/Postprocessed: polished
3	Polyjet	Silicone mat
4	Polyjet	O-ring
5	Polyjet/DLP	Silicone mat & o-ring
6	DLP	Printed sealing mats

**Table 2:** Comparison of different valve designs (DLP: digital light processing).

Manufacturing	Advantages	Disadvantages	Recommended Applications
commercial	+ reliable		• high pressure requirements
3D printed			
radial compression	+ customizable	− no external sealing elements	• custom applications with
	+ small footprint	− adjustment of fit required	flow paths of low complexity
			• low pressure requirements
axial compression	+ customizable	− comparable high footprint	• custom applications with
	+ additional sealing	− additional part (cover)	complex flow paths
	elements		• low to medium pressure
			requirements
printing techniques			
polyjetting	+ planar surface		• valves with large sealing
			surfaces
DLP	+ small channel		• deadvolume is critical
	diameter (<0.8 mm)		

## Data Availability

The data presented in this study are available in the Supplementary Material.

## Supplementary Materials

The following are available online at https://www.mdpi.com/article/10.3390/mi12101247/s1, Figure S1: Microscope images of the sealing surfaces of different valve configurations with 50× magnification; (a) polyjetted valve without postprecessing; (b) polyjetted valve with postprocessing (polished); (c) DLP printed valve; (d) silicone sealing mat, Figure S2: Microscope images of o-rings at magnifications of 50× (a1–c1) and 200x (a2–c2); (a) new o-ring put into the valves o-ring groove; (b) o-ring after 20 valve rotations with sealing concept 5 (silicon mat at opposite sealing surface); (c): o-ring after 20 valve rotations with sealing concept 4 (without silicone mat), Figure S3: In- and outlet flow rates during switching process for the 3D-printed valve connected to the microfluidic flow control system, stl-file S1: Cover of the testvalve, stl-file S2: Rotor of the testvalve, stl-file S3: Rotor of the testvalve with o-ring grooves, stl-file S4: Stator of the testvalve, stl-file S5: 3D-printed sealing mat with o-ring-like structures, txt-file S1: Stepper-motor configuration.

## Abbreviations

The following abbreviations are used in this manuscript:

CAD	Computer-aided design
DLP	Digital light processing
PDMS	Polydimethylsiloxane
