# Supplementary material for: Configurable 3D Printed Microfluidic Multiport Valves with Axial Compression
Source: Micromachines (Basel). 2021 Oct 14;12(10):1247. doi: 10.3390/mi12101247 (PMC8537448; doi:10.3390/mi12101247)
Supplement: Supplementary file 1 [file micromachines-12-01247-s001.zip › SI/SupplementaryMaterials.pdf]

Supplementary Materials

Configurable 3D Printed Microfluidic Multiport Valves with Axial Compression

Juliane Diehm[1], Verena Hackert[1], Matthias Franzreb[1*]

This PDF file includes:

Figure S1

Figure S2

Figure S3

Other Supplementary Materials for this manuscript include the following:

Stl-file S1: Cover of the testvalve

Stl-file S2: Rotor of the testvalve

Stl-file S3: Rotor of the testvalve with o-ring grooves

Stl-file S4: Stator of the testvale

Stl-file S5: 3D-printed sealing matt with o-ring like structures

Txt-file S1: Stepper-motor configuration

[1] Institute of Functional Interfaces, Karlsruhe Institute of Technology, Eggenstein-Leopoldshafen, 76344, Germany; E-Mail: matthias.franzreb@kit.edu

Figure S1 shows microscope images of sealing surfaces of four test valves. Figure S1 a) depicts the sealing surface of a polyjetted valve (sealing concept 1). The surface is quite rough due to circular structures of the printer nozzles, additionally long furrows are visible between the printer heads. Figure S1 b) shows a polished polyjetted part (sealing concept 2). It is evident that the circular structures caused by the printer nozzles were smoothed with polishing, but some of the long furrows are still visible. In addition, small grooves that were probably caused by the polishing with the rotary tool are visible. Though the images only allow for a qualitative estimation of the surface roughness it is obvious that it is reduced with polishing, but the surface is still rough afterwards. For comparison Figure S1 c) shows the surface of a DLP printed valve. It also has some furrows but the surface makes a smoother impression compared to the polyjetted valve. Figure S1 d) shows the surface of the silicone mat that was used as a sealing element. The surface is not as rough as for the 3D printed parts but it is neither completely smooth. This suggests that sealing efficiency not only depends on the surface roughness but also on the flexibility of the material.

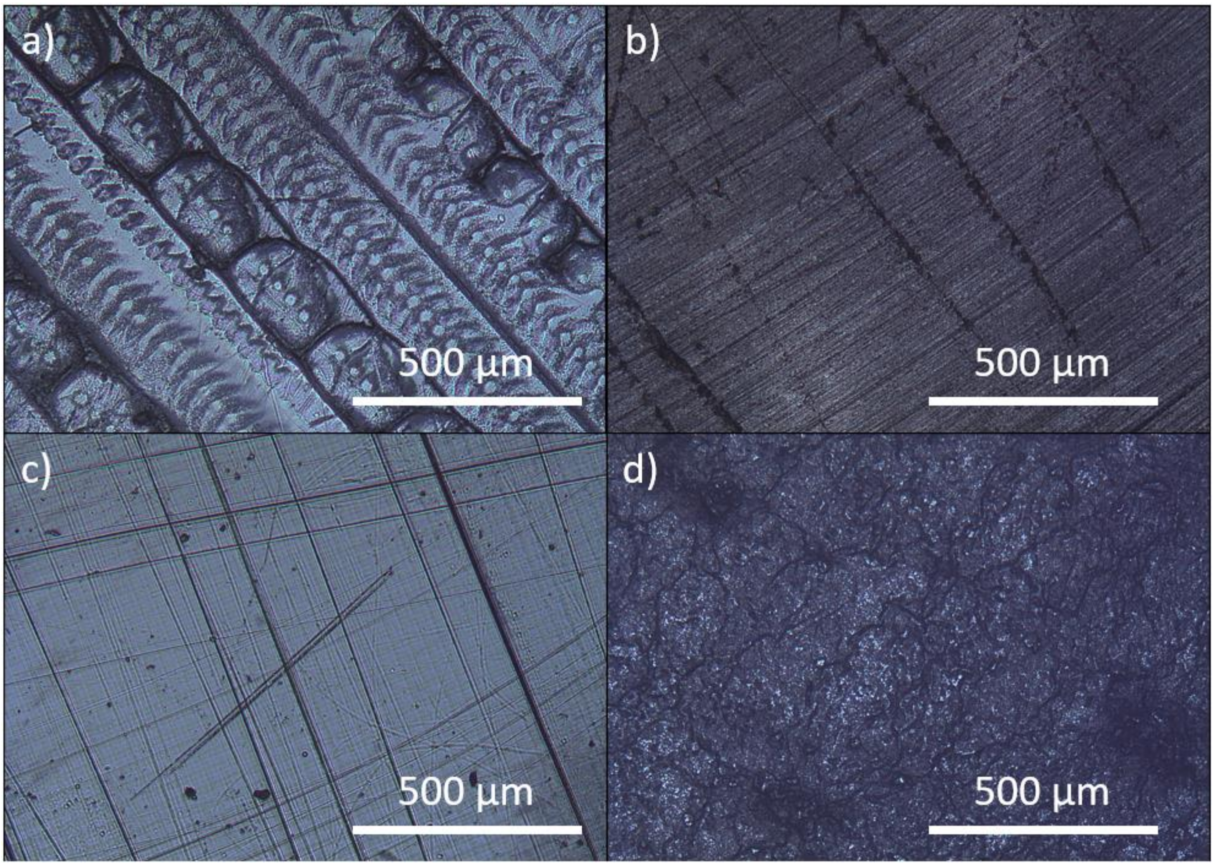

Figure S1 Microscope images of the sealing surfaces of different valve configurations with 50x magnification; a) polyjetted valve without postprecessing; b) polyjetted valve with postprocessing (polished); c) DLP printed valve; d) silicone sealing matt.

Figure S2 shows microscope images of o-rings before and after usage. Figure S2 a) depicts a new o-ring for comparison. Figure S2 b) depicts an o-ring that was rotated 20 times in a valve with a silicone mat on the opposite sealing surface. Comparing the lower magnification (b1) with the original o-ring (a1), it gets clear that the o-ring is slightly deformed by the compression of the valve and is flattened at the surface. Comparing the structure of the material at higher magnification (a1 & b1) no change is visible, thus the o-ring is still intact. Figure S2 c) depicts an o-ring that was rotated 20 times in a valve without a silicone mat on the opposite sealing surface. Figure S2 c1) shows that the o-ring has an uneven surface where it is hard to set the focus level of the microscope and signs of abrasion are visible (white circle). Looking at the material structure (c1) it is obvious that it is less dense compared to the previous two cases with holes of the size of several µm. From these images it can be concluded that the 3D

printed parts are no suitable counter surface for sealing with o-rings but the lifetime can be increased using other counterparts, like silicone mats.

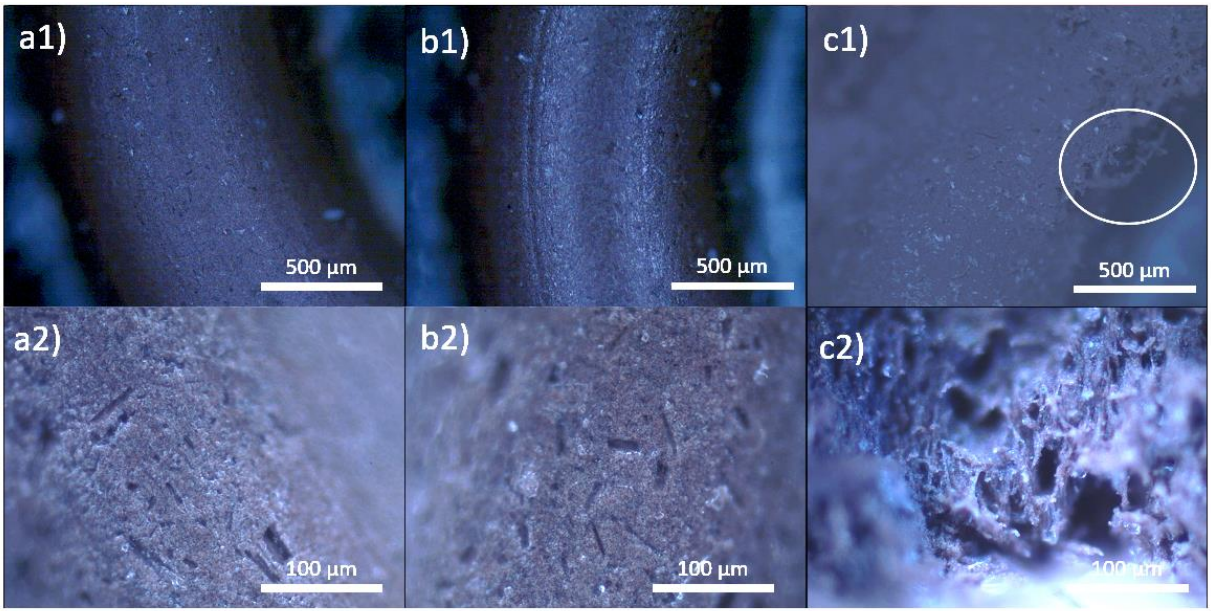

Figure S2 Microscope images of o-rings at maginfications of 50x (a1)-c1)) and 200x (a2-c2)); a) new o-ring put into the valves o-ring groove; b) o-ring after 20 valve rotations with sealing concept 5 (silicon mat at opposite sealing surface); c: o-ring after 20 valve rotations with sealing concept 4 (without silicone mat).

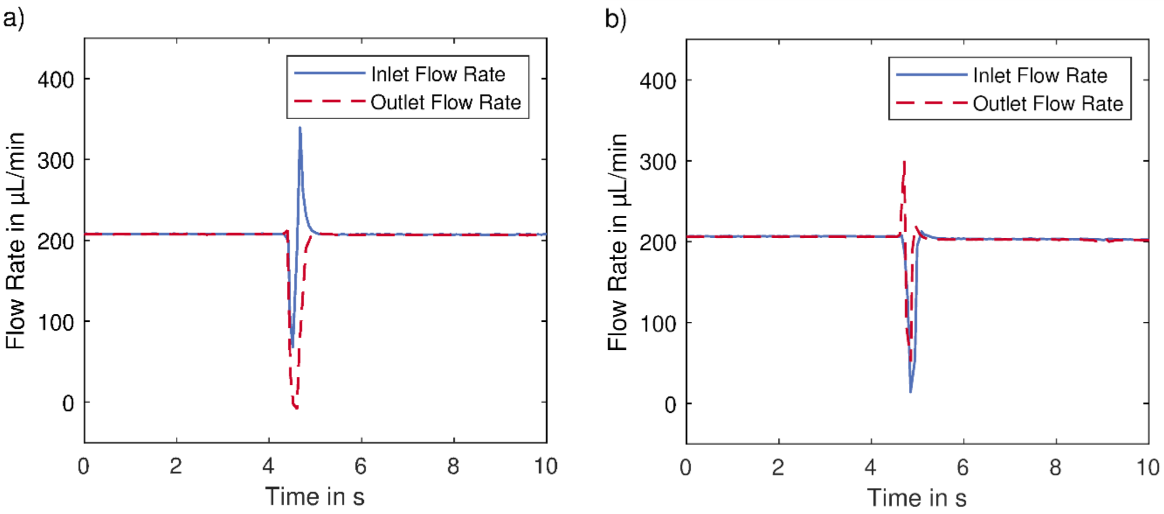

*Figure S3 In- and outlet flow rates during switching process for the 3D printed valve connected to the microfluidic flow control system; (**a**) not tight case with leakage occurring during the switching process; (**b**) tight case with no leakage occurring during the switching process.*
